# Insight into the Photoluminescence of Ba_2_Cd(BO_3_)_2_: RE^3+^ (RE = Dy, Tb) Phosphors

**DOI:** 10.1007/s10895-025-04349-0

**Published:** 2025-05-10

**Authors:** S. B. Töreli, V. E. Kafadar, F. M. Emen, E. Öztürk, R. Altınkaya

**Affiliations:** 1 Department of Materials Science and Engineering, Faculty of Engineering, Adana Alparslan Türkeş Science and Technology University, Adana, 01250 Türkiye; 2 Department of Engineering Physics, Faculty of Engineering, Gaziantep University, Gaziantep, 27310 Türkiye; 3 Faculty of Arts and Sciences, Department of Chemistry, Burdur Mehmet Akif Ersoy University, Burdur, Türkiye; 4 Department of Chemistry, Faculty of Science, Hacettepe University, Ankara, Türkiye; 5 Department of Metallurgical and Materials Engineering, Faculty of Engineering, Karamanoğlu Mehmetbey University, Karaman, Türkiye; 6 Scientific and Technology Application and Research Center, Burdur Mehmet Akif Ersoy University, Burdur, 15030 Türkiye

**Keywords:** Phosphor, Photoluminescence, Inorganic borate, Solid-state reaction, CIE diagram

## Abstract

Dy^3+^ and Tb^3+^ ions doped Ba_2_Cd(BO_3_)_2_ phosphors with varying concentrations (2, 3, 4, 5, 6 mol%) were produced via the solid-state synthesis method in air. The as-synthesized phosphors were characterized. The photoluminescence (PL) and photoluminescence excitation (PLE) spectra of Ba_2_Cd(BO_3_)_2_ phosphors doped with 2, 3, 4, 5, and 6 mol% Dy^3+^ ions reveal four distinct emission bands in the blue, yellow, and red regions, with the 575 nm emission band (^4^F_9/2_ → ^6^H_13/2_, electric dipole transition) exhibiting a notably higher intensity than the 481 nm band (^4^F_9/2_ → ^6^H_15/2_, magnetic dipole transition). The optimal Dy^3+^ doping concentration was identified as 5 mol%, beyond which concentration quenching effects became apparent. Additionally, excitation and emission spectra of Ba_2_Cd(BO_3_)_2_ phosphors doped with 2, 3, 4, 5, and 6 mol% Tb^3+^ ions demonstrate efficient energy absorption at approximately 225 nm, with characteristic emission bands observed at 415, 436, 488, 544, 586, and 621 nm, corresponding to the ^5^D_3_ → ^7^F_5_, ^5^D_3_ → ^7^F_4_, ^5^D_4_ → ^7^F_6_, ^5^D_4_ → ^7^F_5_, ^5^D_4_ → ^7^F_4_, and ^5^D_4_ → ^7^F_3_ transitions, respectively. The ideal concentrations for Dy^3+^ (5 mol%) and Tb^3+^ (6 mol%) in Ba_2_Cd(BO_3_)_2_ are identified at x = 0.3717, y = 0.4064, and x = 0.2902, y = 0.5344, respectively, as per the Commission Internationale de l’Eclairage (CIE) color spectrum, positioning Dy^3+^-doped phosphors within the yellow spectrum and Tb^3+^-doped phosphors within the green spectrum. These phosphors exhibit vibrant yellow and green luminescence, demonstrating their suitability as candidates for applications in these hues. They can be employed when stimulated by near-UV, UV, and blue laser diodes for WLEDs.

## Introduction

Luminescent materials activated by rare-earth ions have been widely explored in displays and lighting, including white light-emitting diodes (WLEDs) and field-emission displays (FEDs). WLEDs have emerged as one of the most promising technologies, driving research due to their high luminous efficiency, brightness, low power consumption, environmental friendliness, and long-term stability, replacing incandescent and fluorescent lamps [1–5]. Fabricating a WLED source can be achieved in two primary ways. The conventional approach combines the yellow phosphor (Y_3_Al_5_O_12_: Ce^3+^ or YAG: Ce^3+^) and an encapsulant organic polymer with an InGaN blue chip to produce white light emission [6]. Since there is no long-wavelength red part in the visible spectrum, these WLEDs have a low color rendering index and high color temperature. With advancements in UV LED research, WLEDs with near-ultraviolet (NUV) chips and red, green, and blue tricolor phosphors can address the mentioned issues [7–10]. The mixture ratios of phosphors can be adjusted to tailor the light source’s chromaticity as needed. The matrix material plays a crucial role in determining the luminescent performance of phosphors. Borate compounds, known for their exceptional chemical and thermal stability, variable crystal field environments, plentiful resource availability, low production cost, moisture resistance, water resistance, and low synthesis temperature, are highly suitable and preferred as matrix materials [11, 12]. Among the rare-earth elements, dysprosium is abundant and widely used in various applications. Dy^3+^-doped phosphors typically produce a white or near-white light due to the unique 4f^9^ electron configuration of Dy^3+^ ions, which effectively absorb UV light. Dy^3+^-activated phosphors have two main emission bands in the blue (470–500 nm) and yellow (560–600 nm) regions, whose relative intensity influences the final chromaticity. Accordingly, the (Y/B) intensity ratio of the phosphor is directly related to white light emission. Since yellow color arises from a hypersensitive transition, it is sensitive to the crystal field environment and can be easily varied. In contrast, Tb^3+^ ions predominantly emit green light (~ 540–550 nm, ^5^D_4_ → ^7^F_5_ transition), with additional weaker emissions in the blue (~ 490 nm) and red (~ 620 nm) regions.

Some recent studies exploring PL properties relying on borate matrix by activating with Dy^3+^ and Tb^3+^ ions searched and revealed the potential of phosphors in solid-state lighting (SSL) applications [13–18]. In this study, a series of yellow and green emitting Ba_2_Cd(BO_3_)_2_: RE^3+^ (RE: Dy, Tb) phosphors were synthesized using the solid-state reaction method. Photoluminescence features of these phosphors are investigated for the first time in the literature. The synthesis of YAG: Ce^3+^ yellow phosphor via the solid-state method necessitates prolonged annealing at elevated temperatures, approximately 1600–1800 °C [19], and Dy^3+^ and Tb^3+^ activated Ba_2_Cd(BO_3_)_2_ phosphors in this study are obtained at comparatively low temperatures at 885 °C, Dy^3+^-doped Ba_2_Cd(BO_3_)_2_ phosphors offer an energy saving and low cost option by replacing the yellow emission, whereas, Tb^3+^ activated Ba_2_Cd(BO_3_)_2_ phosphors provide green components in the production of WLEDs and other solid-state lighting applications. This work is significant as it addresses a critical research gap by synthesizing Ba_2_Cd(BO_3_)_2_: $\:x$Dy^3+^ and Ba_2_Cd(BO_3_)_2_: $\:x$Tb^3+^ (0.02$\:\le\:x\le\:$0.06) phosphors and conducting a systematic investigation of how Dy^3+^ and Tb^3+^ doping affect photoluminescence properties. The study’s novelty lies in its evaluation of these yellow- and green-emitting phosphors for SSL applications, particularly as promising components for WLEDs, contributing to advancements in energy-efficient lighting technologies.

## Experimental Details

### Sample Preparation

Solid-state synthesis route was preferred for the preparation of samples. This cost-effective and versatile technique is particularly suitable for creating phosphors and other advanced functional materials. Its ability to produce high-purity, thermally stable materials with precise stoichiometry, crucial for optimizing properties like luminescence. High-purity reagents of BaCO_3_ (99.9%), Cd(CH_3_COO)_2_.2H_2_O (99.9%), H_3_BO_3_ (99.9%), Dy_2_O_3_ (99.9%), and Tb_2_O_3_ (99.9%) were utilized. To counter B_2_O_3_ evaporation at high temperatures, 6 mol% excess H_3_BO_3_ was added to the starting mixture, achieving a single-phase material. Stoichiometrically proportioned raw materials were weighed, mixed in an agate mortar, and ground for 20 min, with acetone added for homogeneity. The mixtures were then placed in a porcelain crucible and heated in a horizontal tube furnace to 550 °C at 4 °C/min in an air atmosphere, maintained for 10 h, and cooled naturally. After regrounding for 10 min, the samples were reheated to 885 °C at 4 °C/min and sintered in air medium for 10 h before cooling to room temperature. This process yielded Dy^3+^ and Tb^3+^-doped Ba_2_Cd(BO_3_)_2_ powder samples for subsequent structural, morphological, and photoluminescence characterization.

### Characterization

The XRD profiles for undoped, Dy^3+^, and Tb^3+^-doped powders at various concentration levels were obtained using a Rigaku MiniFlex 600 diffractometer with CuKα radiation (λ = 1.5406 Å). The device utilized a 0.02° scanning step in the 2θ range of 10° to 90° at an accelerating voltage of 40 kV and a current of 15 mA. Scherrer’s equation facilitated the calculation of average crystallite sizes. For FT-IR spectroscopy data collection, a Shimadzu IRTracer-100 spectrometer with the KBr method was employed, covering the wavenumber range of 400–4000 cm^− 1^ with a resolution of 4 cm^− 1^ to identify sample functional groups. The surface morphology and chemical composition were examined using a Zeiss Gemini 300 FE-SEM equipped with an EDS detector. The photoluminescence (PL) properties were analyzed using an Agilent Cary Eclipse fluorescence spectrophotometer, with all PL measurements conducted in an open atmosphere at room temperature.

## Results and Discussion

### XRD Studies

**Fig. 1:**
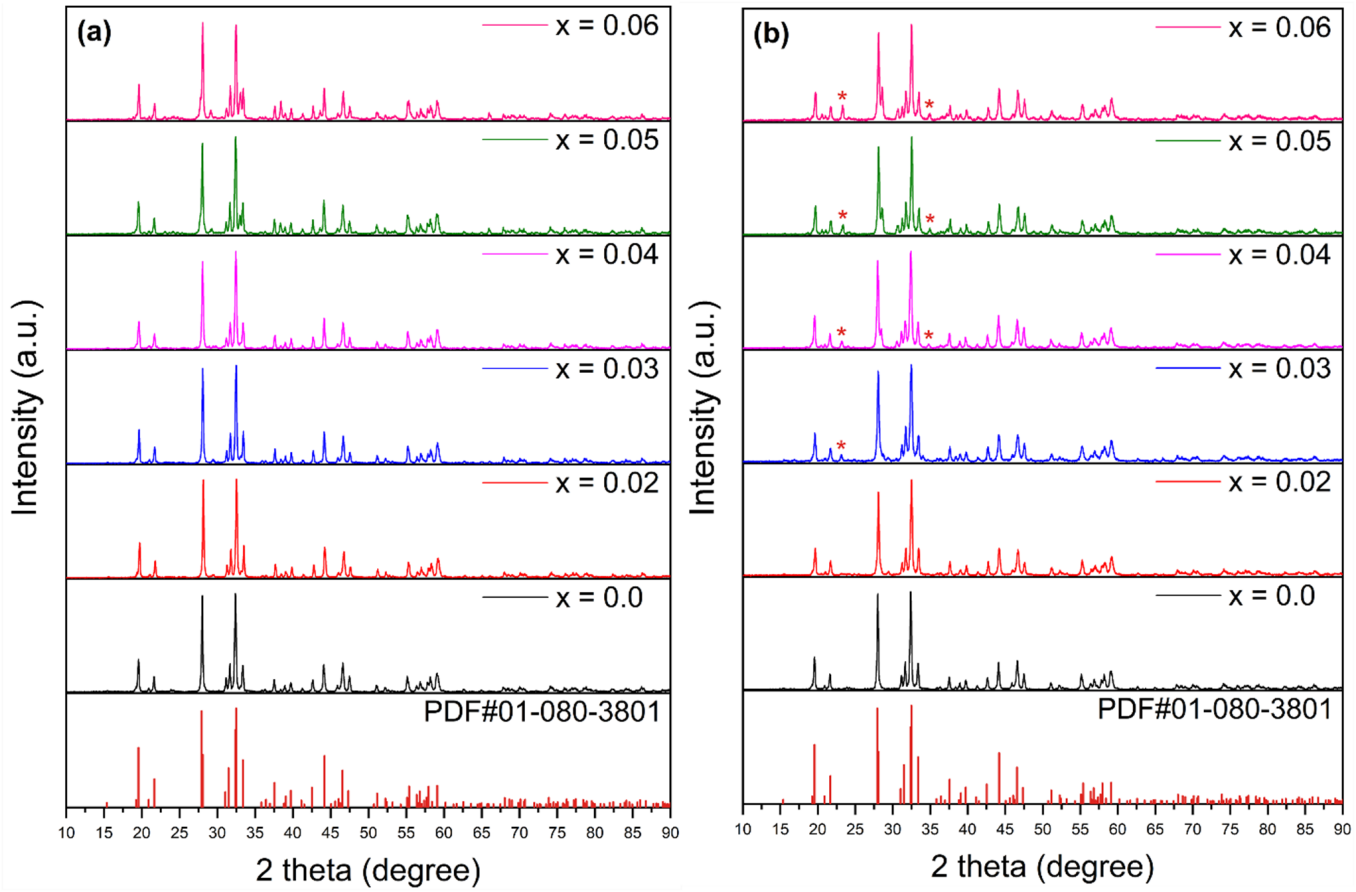
XRD profiles of (**a**) Ba_2_Cd(BO_3_)_2_: $\:x$Dy^3+^ (x = 2, 3, 4, 5, 6 mol%) phosphors and (**b**) Ba_2_Cd(BO_3_)_2_: $\:x$Tb^3+^ (x = 2, 3, 4, 5, 6 mol%) phosphors with Ba_2_Cd(BO_3_)_2_ host and standard card of Ba_2_Cd(BO_3_)_2_ (PDF#01-080-3801)

Figure 1(a) and (b) present the XRD patterns for undoped, Dy^3+^, and Tb^3+^-doped Ba_2_Cd(BO_3_)_2_ phosphor materials. The patterns do not exhibit the presence of compounds such as BaCO_3_, Cd(CH_3_COO)_2_·2H_2_O, H_3_BO_3_, Dy_2_O_3_, Tb_2_O_3_, or other related phases. However, minor impurity peaks appear around 23.0° and 34.5°, corresponding to the BaO phase, as indicated by the asterisk in Fig. 1(b) for the samples with 3, 4, 5, and 6 mol% Tb^3+^ dopings. The detailed structural analysis of this particular borate host, which is Ba_2_Cd(BO_3_)_2_, was first introduced by Zhang et al., and these peaks result from the comparatively high melting point of BaO in the BaO-CdO-B_2_O_3_-Tb_2_O_3_ [20]. Apart from these, the XRD patterns of both the host and Dy^3+^ and Tb^3+^-doped samples align well with the standard card profile PDF#01-080-3801 from the International Centre for Diffraction Data (ICDD) database. The positions of the diffraction peaks stay largely constant with increasing doping of Dy^3+^ and Tb^3+^, indicating the successful incorporation of Dy^3+^ and Tb^3+^ ions and the creation of the intended crystalline compounds in a single phase. The Scherrer formula was used to calculate the size of the crystallites based on the most prominent and well-defined diffraction peak:

$$\:D=\frac{K\lambda\:}{\beta\:\text{cos}\theta\:}$$

In this context, D represents the average crystallite size, K denotes the Scherrer constant (with a value of 0.94), λ is the wavelength of the X-ray source, β corresponds to the full width at half maximum (FWHM) in radians, and θ indicates the diffraction angle. The average crystallite size for the undoped sample is determined to be 38.8 nm. In contrast, the size ranges from 36.2 nm to 42.1 nm for various Dy^3+^-doped samples and from 30.6 nm to 34.3 nm for different Tb^3+^-doped samples.

### FT-IR Studies

**Fig. 2:**
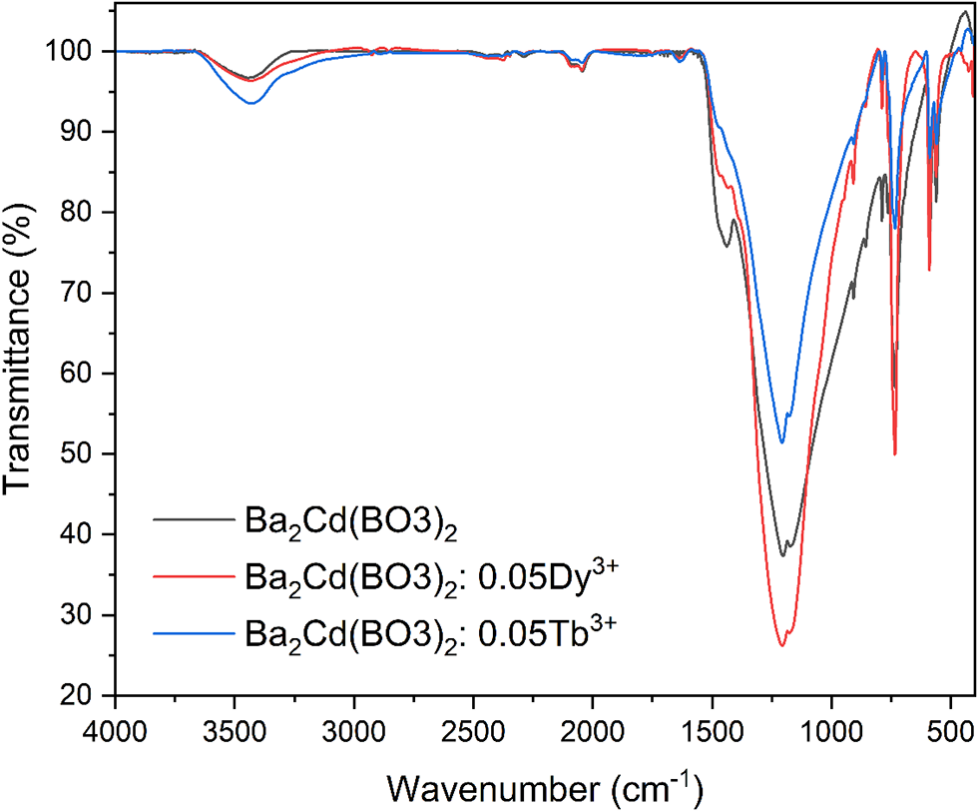
FT-IR spectra of Ba_2_Cd(BO_3_)_2_ host, Ba_2_Cd(BO_3_)_2_: $\:x$Dy^3+^ (x = 5 mol%), and Ba_2_Cd(BO_3_)_2_: $\:x$Tb^3+^ (x = 5 mol%) phosphor

Figure 2 displays the FT-IR spectra of undoped Ba_2_Cd(BO_3_)_2_, Ba_2_Cd(BO_3_)_2_: 0.05Dy^3+^, Ba_2_Cd(BO_3_)_2_: 0.05Tb^3+^, which were obtained to validate the presence of structural units in the host material and doped materials. Absorption peaks between 1000 and 1600 cm^− 1^ are caused by the lattice vibrations of the BO_3_ structural networks in borate materials. The pronounced absorption peaks at 1205 cm^− 1^ and comparatively feeble absorption peaks at 1438 cm^− 1^ are ascribed to the B-O asymmetric stretching vibration mode of the triangular [BO_3_]^3−^ units [21]. The spectral bands in the 600–800 cm^− 1^ range, peaking at 734 cm^− 1^, can be attributed to the B-O out-of-plane bending vibrations of the BO_3_ units [22]. This observation also confirms the presence of [BO_3_]^3−^ groups within the structures. Additionally, the band observed at 908 cm^− 1^ is associated with the symmetric stretching vibrations of the BO_3_ unit, while the bands at 856 cm^− 1^ correspond to the vibrations of Ba-O bonds [22, 23]. The spectra demonstrate a distinct absorption band at 589 cm^− 1^, associated with the in-plane bending vibration of O-B-O within the borate network [24]. The bands noted below 589 cm^− 1^ predominantly arise from the lattice dynamic modes [25]. The replacement of Dy^3+^ and Tb^3+^ at the Ba site in Ba_2_Cd(BO_3_)_2_ resulted in a little shift of 3–4 cm^− 1^ in the FT-IR absorption band, suggesting the dopants do not significantly change the basic structure of the borate. The results confirm the existence of solely trigonally coordinated boron atoms in the produced phosphors, consistent with the properties of compounds featuring BO_3_ functional groups as reported in the literature [26].

### FE-SEM Images with EDS Analysis

**Fig. 3:**
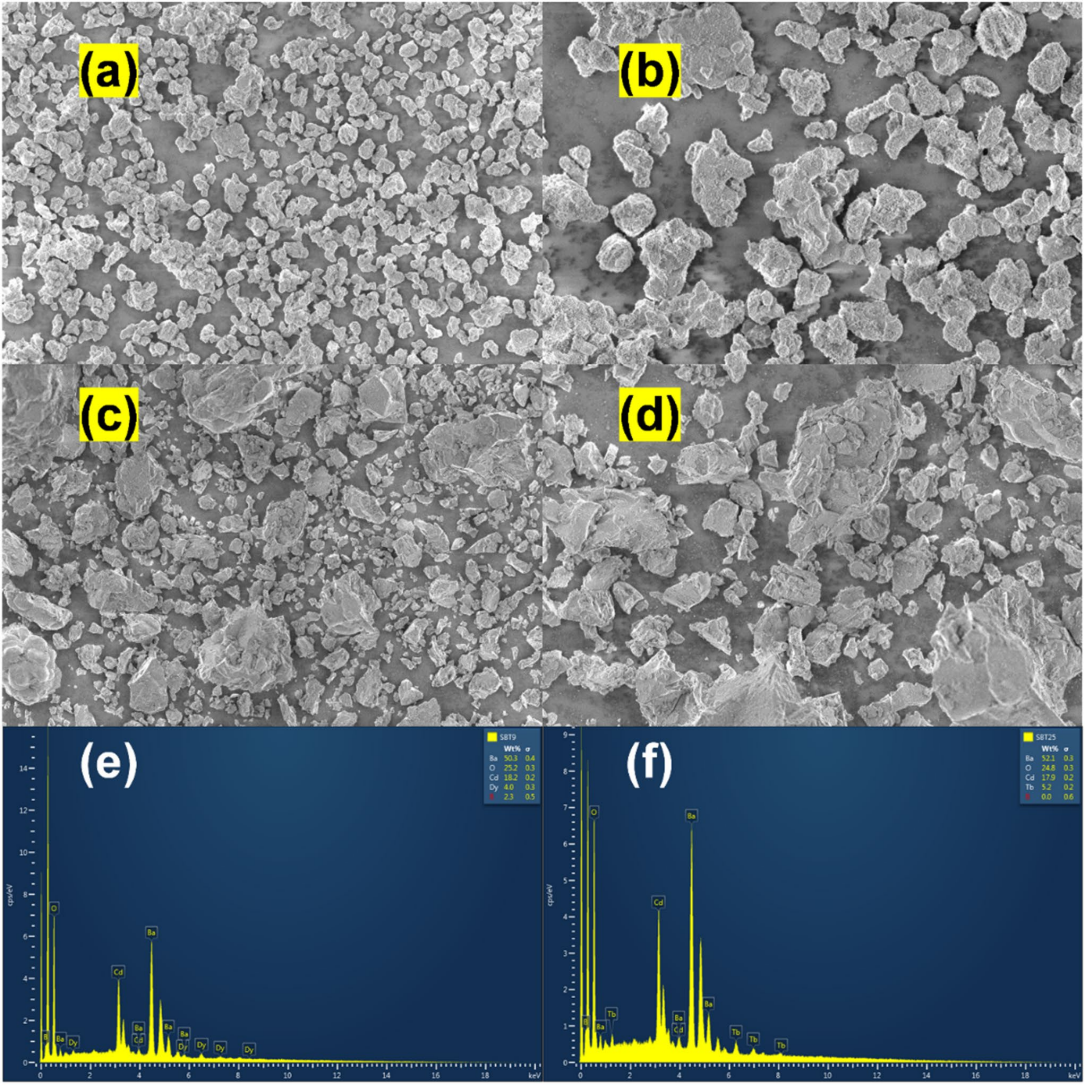
FE-SEM images at (**a**) 2000x, (**b**) 5000x magnification with (**e**) EDS spectrum for Ba_2_Cd(BO_3_)_2_: $\:x$Dy^3+^ (x = 5 mol%) phosphor, and (**c**) 2000x, (**d**) 5000x magnification with (**f**) EDS spectrum for Ba_2_Cd(BO_3_)_2_: $\:x$Tb^3+^ (x = 5 mol%) phosphor

Figure 3(a-d) illustrates the sample morphology via FE-SEM micrographs at varying magnifications (2000x, 5000x) for the 5 mol% Dy^3+^ and 5 mol% Tb^3+^-doped Ba_2_Cd(BO_3_)_2_ phosphors. The irregularly shaped particles are predominantly observed throughout the surface. Dy^3+^-doped sample mainly features small-sized particles uniformly dispersed across the surface, with no obvious aggregation. As for the Tb^3+^-doped sample, it presents an uneven distribution of particles of varying sizes on its surface. Figure 3(e, f) depicts the EDS spectrum, highlighting the presence of all requisite elements, while the insets in Fig. 3(e, f) illustrate the estimated elemental percentages of Ba, Cd, B, O, Dy, and Tb. The performed EDS scan to analyze the chemical compositions confirms the presence of Dy^3+^ and Tb^3+^ ions in Ba_2_Cd(BO_3_)_2_. The atomic percentages obtained for Dy^3+^ (4.0 at%) and Tb^3+^ (5.2 at%) dopants closely approximate the stoichiometric ratio of both 5 mol% Dy^3+^ and Tb^3+^-doped Ba_2_Cd(BO_3_)_2_. EDS profiles indicate the presence of just Ba, Cd, B, O, Dy, and Tb elements in the sample, with no detectable impurity elements. This outcome corroborates the phase purity of the synthesized phosphors and aligns well with the structural analysis.

### PL Studies

#### Ba_2_Cd(BO_3_)_2_: Dy^3+^

**Fig. 4:**
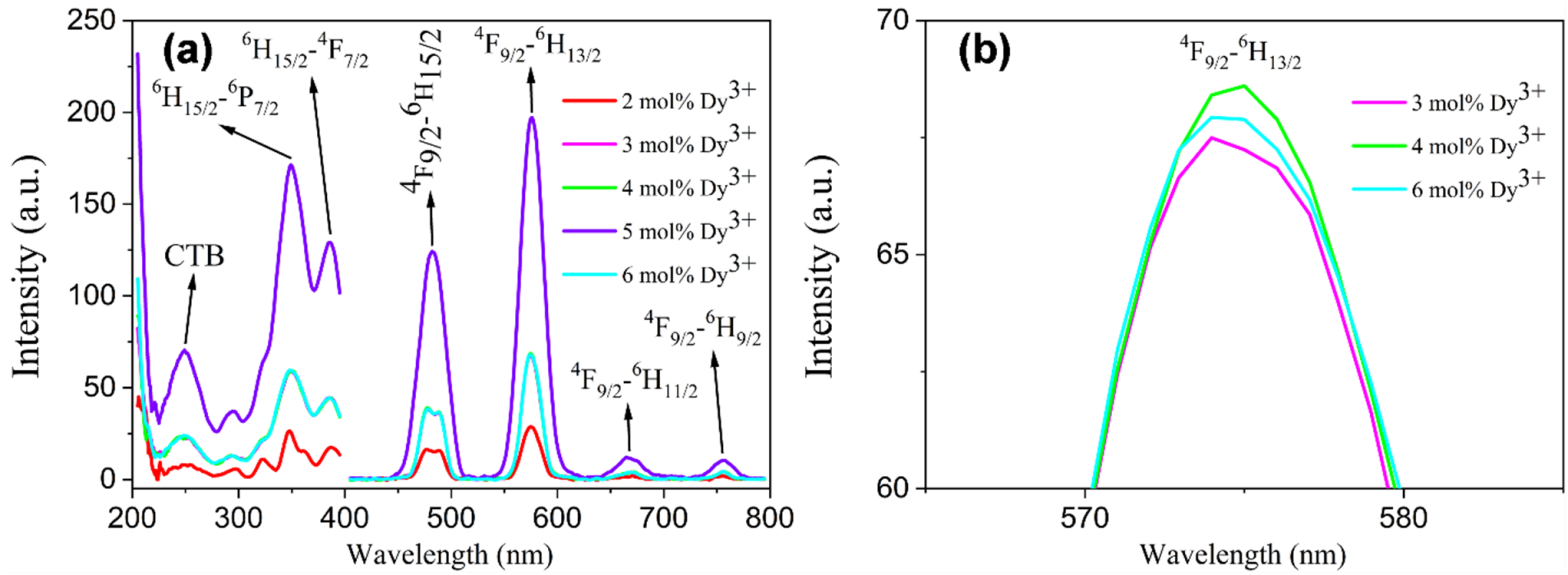
(**a**) PLE and PL spectra of Ba_2_Cd(BO_3_)_2_: $\:x$Dy^3+^ (x = 2, 3, 4, 5, 6 mol%) phosphors at 200–800 nm, (**b**) PL spectra of Ba_2_Cd(BO_3_)_2_: $\:x$Dy^3+^ (x = 3, 4, 6 mol%) at 570–580 nm

The photoluminescence excitation and emission spectra of the 2, 3, 4, 5, and 6 mol% Dy^3+^ ions doped Ba_2_Cd(BO_3_)_2_ phosphors are presented in Fig. 4. The characteristic excitation bands resulting from the ^6^H_15/2_ → ^6^P_7/2_ and ^6^H_15/2_ → ^4^F_7/2_ transitions at 350 nm and 386 nm of Dy^3+^ ions are observed [27, 28]. Among these, the excitation band at 350 nm causes the most intense excitation. For this reason, we can say that the excitation energy is mainly captured by Dy^3+^ ions. Also, the charge transfer band (CTB) transition at 250 nm causes an ultraviolet excitation in all phosphors. Therefore, 2, 3, 4, 5, and 6 mol% Dy^3+^ ions doped Ba_2_Cd(BO_3_)_2_ phosphors are very suitable candidates for excited blue laser diodes or UV/near-UV. The contribution of Dy^3+^ ions to the luminescence mechanism varies depending on whether they are used as activator ions or co-activator ions [29, 30]. At a monitoring wavelength of 350 nm, the emission spectrum (Fig. 4) consists of characteristic emission bands of Dy^3+^ ions at blue, yellow, and red regions. The emission spectra show the characteristic emission peaks located at 481, 575, 667, and 757 nm, corresponding to the ^4^F_9/2_→^6^H_15/2_, ^4^F_9/2_ → ^6^H_13/2_, ^4^F_9/2_ → ^6^H_11/2,_ and ^4^F_9/2_ → ^6^H_9/2_ transitions of Dy^3+^ ions, respectively [27, 28, 31–34]. The ^4^F_9/2_ → ^6^H_15/2_ transition of Dy^3+^ ions at 481 nm is a magnetic dipole transition and is independent of and unaffected by the coordination environment of the Dy^3+^ ions in the host crystal. The ^4^F_9/2_ → ^6^H_13/2_ transition, which is the most intense emission at 575 nm, is an electric dipole transition that is allowed only at low symmetries with no inversion center and is extremely sensitive to the coordination environment of the Dy^3+^ ions in the host crystal [28, 33]. The luminescence intensity of the ^4^F_9/2_ → ^6^H_13/2_ transition is higher than that of the ^4^F_9/2_ → ^6^H_15/2_ transition. For this reason, it is seen that Dy^3+^ ions in Ba_2_Cd(BO_3_)_2_ phosphors are predominantly located in low symmetry positions. When the doping ratios of Dy^3+^ ions and emission intensity of the ^4^F_9/2_ → ^6^H_13/2_ transition are evaluated together, it is seen that the emission intensity increases as the doping ratio increases, reaches a maximum at a 5% doping ratio, and decreases due to concentration quenching when the doping ratio is 6 mol% (Fig. 4b). Concentration quenching is observed in the PL study with 6 mol% Dy^3+^-doped sample due to interactions between activator ions when their concentration exceeds a certain threshold. At higher dopant concentrations, activator ions such as Dy^3+^ are positioned closer together, leading to non-radiative energy transfer between these ions. This transfer reduces the overall radiative emission efficiency. Therefore, the optimal concentration of Dy^3+^ ions was determined to be 0.05. The concentration quenching may occur as a result of non-radiative energy transfer between Dy^3+^ ions. This energy transfer is caused by multipole-multipole interaction, radiation reabsorption, and exchange interaction [35].

#### Ba_2_Cd(BO_3_)_2_: Tb^3+^

**Fig. 5:**
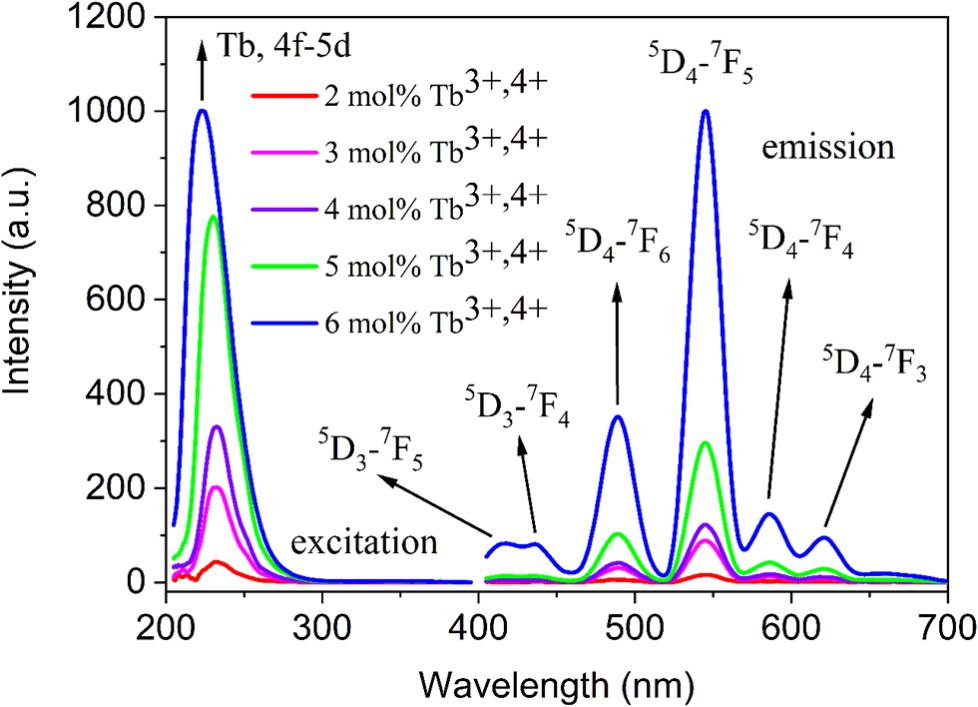
PLE and PL spectra of Ba_2_Cd(BO_3_)_2_: $\:x$Tb^3+^ (x = 2, 3, 4, 5, 6 mol%) phosphors at 200–800 nm

In order to obtain the radiation of Tb^3+^ ions at different wavelengths, different ratios of Tb^3+^ ions were doped into the Ba_2_Cd(BO_3_)_2_ housing crystal. Figure 5 shows the photoluminescence spectra of Ba_2_Cd(BO_3_)_2_ doped with 2, 3, 4, 5, and 6 mol% Tb^3+^ ions. As seen in the excitation spectrum, there is a single broad excitation band in the 200–300 nm range for all doping ratios. It is seen that the excitation energy is captured at the same wavelength maximum at 2, 3, 4, and 5% doping ratios [36, 37]. When the doping ratio is 6%, it is seen that the maximum of the excitation wavelength slightly shifts towards the lower wavelength. It is understood that the excitation energy causes the 4f-5d transition of Tb^3+^ ions for all doping ratios. It is seen that the excitation intensity increases regularly as the doping ratio rises regularly. The excitation band of phosphors shows that 2, 3, 4, 5, and 6 mol% Tb^3+^ ions doped Ba_2_Cd(BO_3_)_2_ are extremely suitable candidates for the design of phosphors excited by blue laser diodes or UV/near-UV.

The emission spectra of materials doped with 2, 3, 4, 5, and 6 mol% Tb^3+^ are shown in Fig. 5. Focusing on the doping ratio of 2, 3, 4, and 5%, the emission bands of Tb^3+^ ions at 488, 544, 586, and 621 nm for the ^5^D_4_ → ^7^F_6_, ^5^D_4_ → ^7^F_5_, ^5^D_4_ → ^7^F_4_, and ^5^D_4_ → ^7^F_3_ transitions are obtained [34, 38–41]. Blue, green, yellow, and red region emissions are observed in the emission spectrum, and no emission bands belonging to the blue region are observed before 488 nm. However, when the doping ratio is increased and 6 mol% Tb^3+^ ions are doped, the emission of Tb^3+^ doped Ba_2_Cd(BO_3_)_2_ is broadened. At this doping rate, in addition to the emission bands of ^5^D_4_ → ^7^F_6_, ^5^D_4_ → ^7^F_5_, ^5^D_4_ → ^7^F_4_ and ^5^D_4_ → ^7^F_3_ transitions of Tb^3+^ ions at 488, 544, 586, and 621 nm, the emission bands originating from ^5^D_3_ → ^7^F_5_, ^5^D_3_ → ^7^F_4_ transitions at 415 and 436 nm are obtained. We can easily say that when the doping rate of Tb^3+^ ions in the Ba_2_Cd(BO_3_)_2_ host crystal is 6%, this phosphor material also gains the additional blue region emission, and the obtained material is now a material that emits in the blue, green, yellow, and red regions. The difference between the ^5^D_3_ and ^5^D_4_ energy levels of Tb^3+^ is quite small. Therefore, the ^5^D_4_ level is efficiently filled by non-radiative relaxation [38]. As seen in the emission spectrum, the most intense emission peak is due to the ^5^D_4_ → ^7^F_5_ transition of Tb^3+^ ions at 544 nm. This transition is called the magnetic dipole transition of Tb^3+^ ions at the ΔJ = 1 state, while the second most intense transition, the ^5^D_4_→^7^F_6_ transition, is of electric-dipole nature [42]. Similar to the radiation intensity, as the doping rate of Tb^3+^ ions increases, the emission intensity also increases. When both excitation and radiation intensity are considered together, the optimum doping rate is 6 mol%.

### CIE, CCT, and Color Purity

**Fig. 6:**
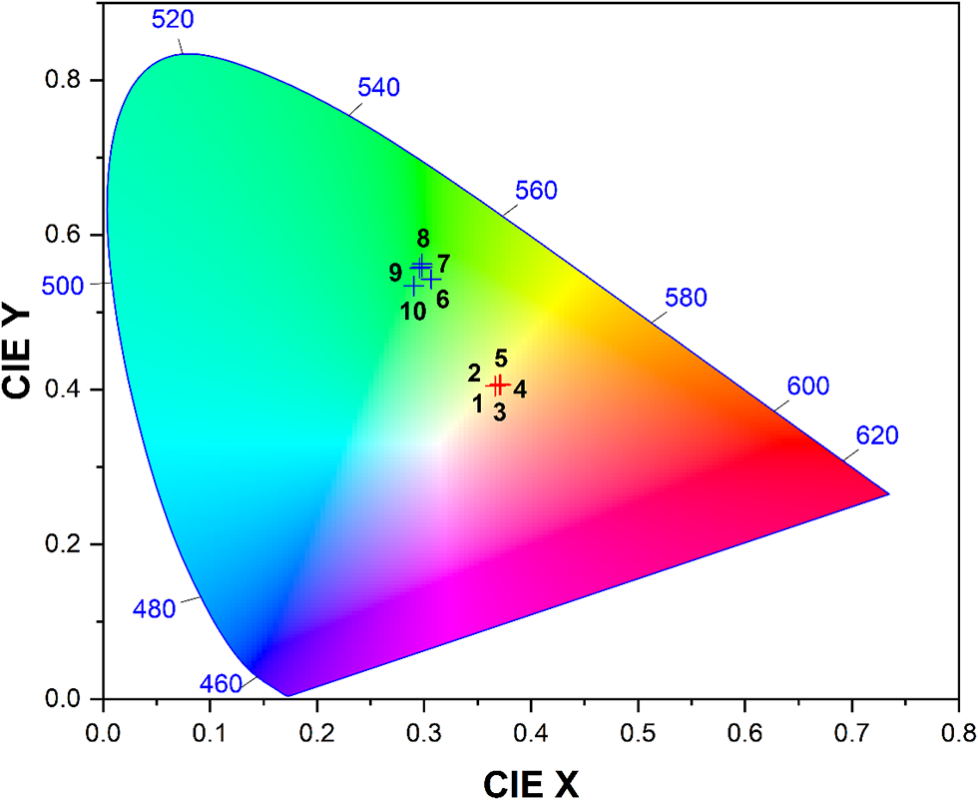
CIE chromaticity diagram of the Ba_2_Cd(BO_3_)_2_: $\:x$Dy^3+^ and Ba_2_Cd(BO_3_)_2_: $\:x$Tb^3+^ (where x = 2, 3, 4, 5, 6 mol%) phosphors

To analyze the color coordinates of Ba_2_Cd(BO_3_)_2_: Dy^3+^ and Ba_2_Cd(BO_3_)_2_: Tb^3+^ phosphors, a scientific graphing software incorporating the CIE-1931 color chromaticity diagram template was employed. Table 1 details the determined chromaticity coordinates (x, y) of the Ba_2_Cd(BO_3_)_2_: Dy^3+^ and Ba_2_Cd(BO_3_)_2_: Tb^3+^ phosphors with 2, 3, 4, 5, and 6 mol% dopings. The optimal concentration for Dy^3+^ (5 mol%) and Tb^3+^ (6 mol%) in Ba_2_Cd(BO_3_)_2_ is found at x = 0.3717, y = 0.4064, and x = 0.2902, y = 0.5344, respectively, according to the CIE color spectrum, which places Dy^3+^-doped phosphors in the yellow area and Tb^3+^-doped phosphors in the green region. These phosphors exhibit vivid yellow and green emission. CIE results indicate their potential as candidates for applications desired in these colors. They can be utilized when excited by near-UV, UV, and blue laser diodes for WLEDs (Fig. 6).

**Table 1:** CIE chromaticity coordinates, CCT values, color purity values, and the dominant emission wavelengths of Ba_2_Cd(BO_3_)_2_: $\:x$Dy^3+^ and Tb^3+^ (x = 2, 3, 4, 5, 6 mol%) phosphors

Corresponded point numbers in the CIE diagram	Phosphors	Excitation wavelength (nm)	CIE chromaticity coordinates		CCT (K)	Color purity (%)	Dominant wavelength (nm)
			x	y			
1	2 mol% Dy^3+^	350	0.3665	0.4048	4525	39.65	575
2	3 mol% Dy^3+^		0.3706	0.4066	4426	41.32	575
3	4 mol% Dy^3+^		0.3705	0.4063	4426	41.00	575
4	5 mol% Dy^3+^		0.3717	0.4064	4395	41.22	575
5	6 mol% Dy^3+^		0.3713	0.4069	4410	41.32	575
6	2 mol% Tb^3+^	232	0.3067	0.5430	6022	56.22	546
7	3 mol% Tb^3+^		0.2981	0.5588	6170	59.27	545
8	4 mol% Tb^3+^		0.2981	0.5623	6164	60.00	545
9	5 mol% Tb^3+^		0.2958	0.5572	6221	58.81	545
10	6 mol% Tb^3+^	223	0.2902	0.5344	6392	53.33	545

## Conclusion

In summary, a series of Dy^3+^ and Tb^3+^ single-doped Ba_2_Cd(BO_3_)_2_ phosphors were successfully prepared through the solid-state reaction. XRD patterns of the Dy^3+^ and Tb^3+^-doped samples prove that the compounds are formed and well-indexed to the main PDF card. Photoluminescence spectra of 2, 3, 4, 5, and 6 mol% Dy^3+^ ions-doped Ba_2_Cd(BO_3_)_2_ materials are considered; those materials emit predominantly yellow light under the excitation of 350 nm. The excitation bands showed they can be effectively excited with UV, near-UV, and blue laser diodes. The experimental PL results confirmed that Dy^3+^ ions-doped Ba_2_Cd(BO_3_)_2_ materials have blue, yellow, and red emission regions, and the phosphors are useful for lighting and opto-electronic applications under n-UV and blue laser diode excitation. The excitation and emission spectra shows that the 2, 3, 4, 5, and 6 mol% Tb^3+^ ions doped Ba_2_Cd(BO_3_)_2_ materials capture the excitation energy at about 225 nm and have emission bands at 415, 436, 488, 544, 586, and 621 nm corresponding to ^5^D_3_ → ^7^F_5_, ^5^D_3_ → ^7^F_4_, ^5^D_4_ → ^7^F_6_, ^5^D_4_ → ^7^F_5_, ^5^D_4_ → ^7^F_4_, and ^5^D_4_ → ^7^F_3_ transitions, respectively. Therefore, Ba_2_Cd(BO_3_)_2_ phosphor materials doped with Tb^3+^ ions can be effectively excited with UV, near-UV, and blue laser diodes. Tb^3+^ ions doped Ba_2_Cd(BO_3_)_2_ phosphors have blue, green, yellow, and red emission regions, and the phosphors are extremely suitable material for the mainly green region optoelectronic applications that are excited by UV, near-UV, and blue laser diodes and WLEDs.

## Data Availability

No datasets were generated or analysed during the current study.
